# Data on the expression of CXCR3 ligands and pro-inflammatory cytokines in macrophages and CD4^+^ T cells after stimuli of CXCR3 ligands

**DOI:** 10.1016/j.dib.2018.03.042

**Published:** 2018-03-15

**Authors:** Bongjun Kim, Jong-Ho Lee, Won Jong Jin, Hong-Hee Kim, Hyunil Ha, Zang Hee Lee

**Affiliations:** a Department of Cell and Developmental Biology, Dental Research Institute, School of Dentistry, Seoul National University, Seoul 110-749, Republic of Korea; b The University of Texas MD Anderson Cancer Center, Department of Neuro-Oncology, Huston, TX 77030, USA; c Clinical Research Division, Korea Institute of Oriental Medicine, 483 Expo-Ro, Yuseong-Gu, Daejeon 305-811, Republic of Korea

**Keywords:** CXCR3, CXCL9, CXCL11, Cytokine

## Abstract

C-X-C motif chemokine receptor 3 (CXCR3) is a G protein-coupled receptor for three ligands which are C-X-C motif chemokine 9 (CXCL9), CXCL10, and CXCL11 [1]. Previously we have reported that CXCL10 promotes pro-inflammatory cytokine expression, and forms positive feedback loop [2], [3]. In the present study, we described mRNA expression of CXCL9 and CXCL11 under CXCL10 stimuli in the presence or absence of CXCR3 antagonist, JN-2 in bone marrow-derived macrophages (BMMs) and CD4^+^ T cells. In addition, we examined pro-inflammatory cytokine expression under CXCL9 or CXCL11 stimuli in BMMs and CD4^+^ T cells.

**Specifications Table**

**Table:** 

Subject area	*Biology*
More specific subject area	*Chemokine biology*
Type of data	*Figure, Graph*
How data was acquired	*Real-time PCR*
Data format	*Analyzed*
Experimental factors	*Bone marrow cells were obtained by flushing tibiae and femora of mice and non-adherent cells were expanded for three days using M-CSF to generate bone marrow derived macrophages. CD4*^*+*^*T cells were obtained from spleen of mice using CD4*^*+*^*T cell isolation kit, and activated for three days using anti-CD3 antibody, anti-CD28 antibody, and IL-2.*
Experimental features	*Serum starved Bone marrow derived macrophages and CD4*^*+*^*T cells were cultured with DMSO or JN-2 in the presence or absence of CXCL10 for six hours, and then mRNA expression of CXCL9 and CXCL11 was analyzed by real-time PCR. Serum starved Bone marrow derived macrophages and CD4*^*+*^*T cells were cultured with or without CXCL9 or CXCL11 for 6 hours then mRNA expression of Tnfα, Il6, and Tnfsf11 was analyzed by real-time PCR*
Data source location	*Seoul, Republic of Korea*
Data accessibility	*Data with this article*

**Value of the data**

• In our earlier report, C-X-C motif chemokine 10 (CXCL10) was auto-amplified through NFκB [3]. Data in this study described mRNA expression of CXCL9 and CXCL11 by CXCL10 stimuli.
• In our earlier report, expression of inflammatory cytokine was increased by CXCL10 [3]. Data in this study described the effect of CXCL9 and CXCL11 on mRNA expression of inflammatory cytokine.
• These data provide different gene expression patterns by CXCL9, CXCL10, and CXCL11 which are ligands sharing same receptor.
• Other researchers can consult this data as an example for the effect of different ligands sharing the same receptor.

## 1 Data

**Fig. 1:**
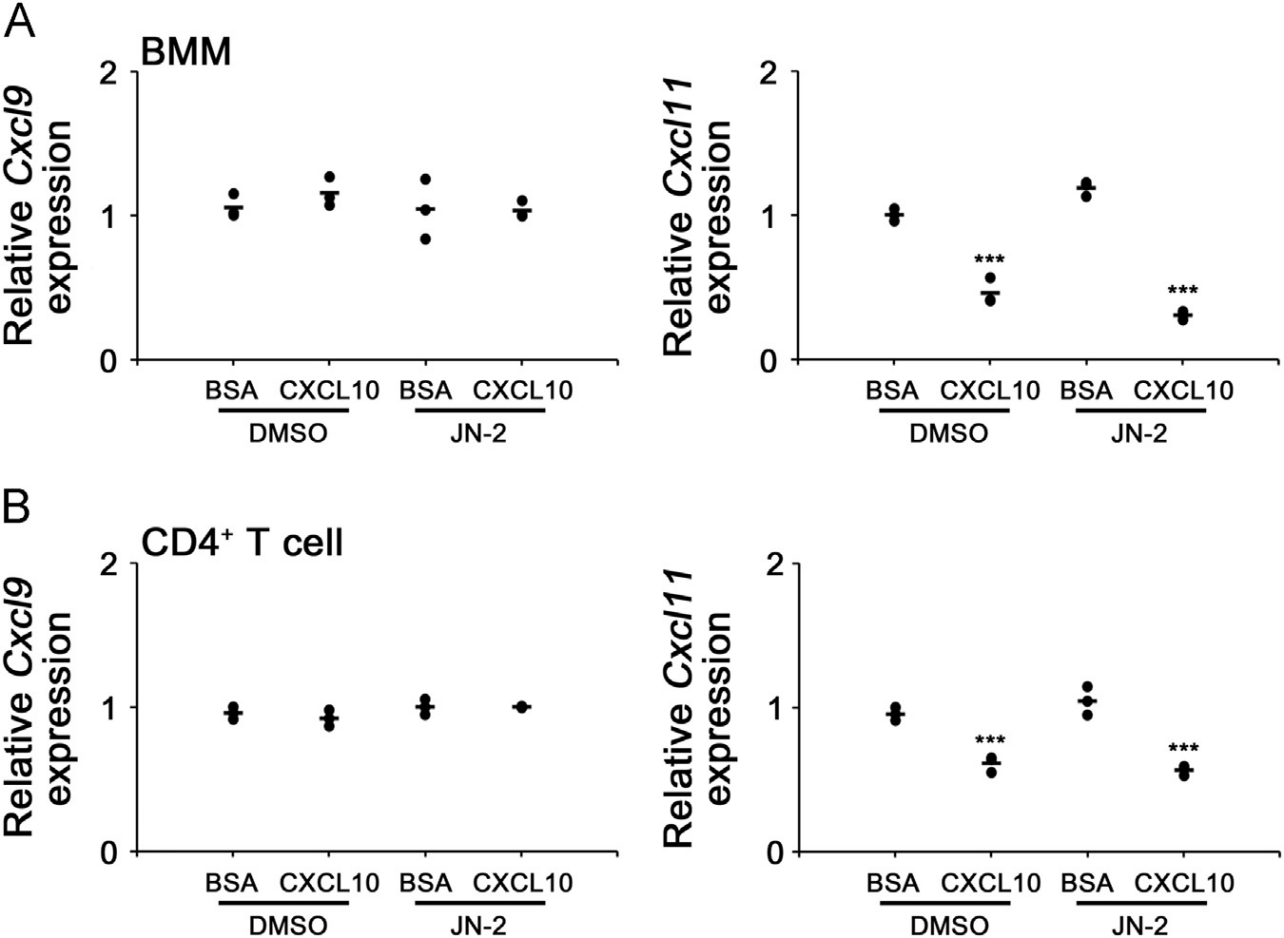
CXCL10 differently regulates mRNA expression of CXCL9 and CXCL11. (A, B) Serum-starved BMMs and CD4^+^ T cells were cultured with or without CXCL10 (100 ng/mL) in the presence of DMSO or JN-2 (25 μM) for 6 h. (A) *Cxcl9* and *Cxcl11* mRNA level in BMMs, was analyzed by real-time PCR. (B) *Cxcl9* and *Cxcl11* mRNA level in CD4^+^ T cells, was analyzed by real-time PCR. β-actin was used as an internal control for normalization for real-time analysis. The results shown are representative of three independent experiments (*n* = 3), and the values are expressed as mean. ***P < 0.001 *vs* each CXCL10-untreated cells by unpaired Student's *t* test.

**Fig. 2:**
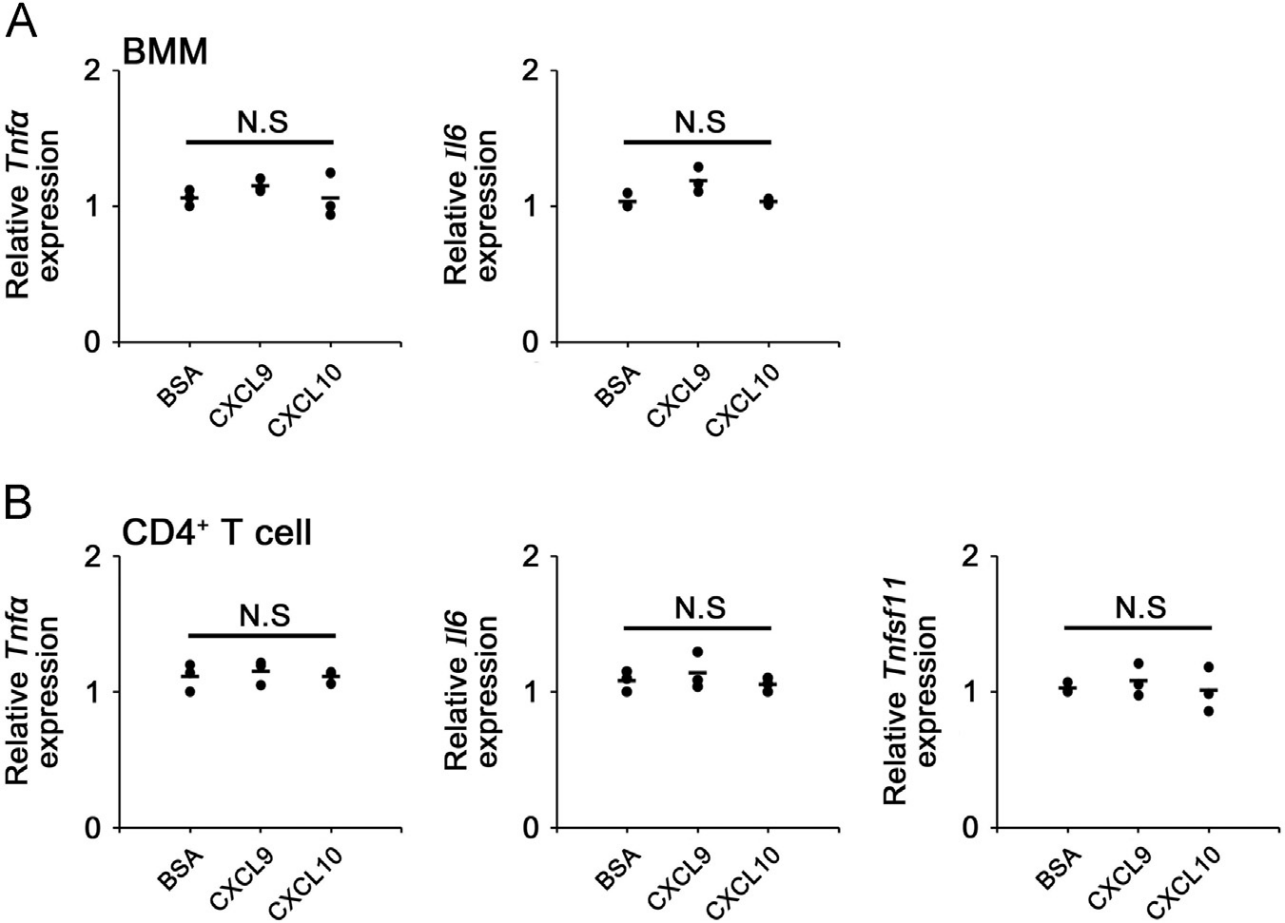
CXCL9 and CXCL11 does not affect expression of inflammatory cytokines. (A, B) Serum-starved BMMs and CD4^+^ T cells were cultured with BSA, CXCL9 (100 ng/mL), or CXCL11 (100 ng/mL) for 6 h. (A) *Tnfα* and *Il6,* and *Tnfsf11* mRNA level in BMM was analyzed by real-time PCR. (B) *Tnfα, Il6,* and *Tnfsf11* mRNA level in CD4^+^ T cell was analyzed by real-time PCR. β-actin was used as an internal control for normalization for real-time analysis. The results shown are representative of three independent experiments (*n* = 3), and the values are expressed as mean ± SD. N.S, not significant.

These data presented mRNA expression of CXCL9 (Cxcl9) and CXCL11 (Cxcl11) under CXCL10 stimuli in the presence or absence of CXCR3 antagonist, JN-2, in BMMs and CD4+ T cells, analyzed by real-time PCR (Fig. 1). The mRNA expression of tumor necrosis factor alpha (TNFa; *Tnfα*), interleukin 6 (IL-6; *Il6*), and receptor activator of nuclear factor kappa-B ligand (RANKL; *Tnfsf11*) under CXCL9 or CXCL11 stimuli, was also analyzed by real-time PCR (Fig. 2).

## 2 Experimental design, materials and methods

### 2.1 Reagents

The CXCR3 antagonist *N*-(4-(5-chlorobenzo[d]oxazol-2-ylamino) phenyl)-4-aminobutanamide (JN-2) was synthesized and purified as described previously [4]. Recombinant mouse CXCL9, CXCL10, CXCL11, and M-CSF were purchased from PeproTech (London, UK).

### 2.2 Cell culture

Bone marrow–derived macrophages (BMMs) were prepared as described previously [5]. In brief, mouse bone marrow cells were obtained from femurs and tibias and incubated in α-minimal essential medium (α-MEM; Welgene, Daegu, Korea) complete media containing 10% fetal bovine serum (FBS), 100 units/mL penicillin, and 100 µg/mL streptomycin on 10-cm culture dishes in the presence of M-CSF (10 ng/mL) overnight. Nonadherent cells were transferred to 10-cm bacterial culture dishes and further cultured in the presence of M-CSF (30 ng/mL) for 3 days. Adherent cells were used as BMMs after the nonadherent cells were washed out.

CD4^+^ T cells were isolated from mouse spleens as described previously [5]. Briefly, spleens were mashed in Hanks’ balanced salt solution containing 3× antibiotics. Cells were harvested, and red blood cells were removed by ammonium-chloride-potassium lysing buffer. The remaining cells were collected and separated with a Ficoll-Histopaque (Sigma, St Louis, MO, USA) discontinuous gradient. The interface containing the cells was washed with PBS and resuspended in CD4^+^ T cell isolation buffer. Resuspended CD4^+^ T cells were purified by negative selection by using CD4^+^ T cell isolation kits (Miltenyi Biotec, Bergisch Gladbach, Germany) according to the manufacturer's instructions. After isolation, CD4^+^ T cells were cultured in RPMI 1640 (Welgene, Daegu, Korea) containing 10% FBS and activated using anti-CD28 antibody (2 μg/mL; eBioscience, San Diego, CA, USA) and interleukin-2 (IL-2; 50 ng/mL; PeproTech, London, UK) on the plate coated with anti-CD3 antibody (2 μg/mL; eBioscience, San Diego, CA, USA) for 3 days.

### 2.3 Reverse transcription and real-time polymerase chain reaction analysis

Total RNA was prepared from cells or spleens by using an RNeasy Mini kit (Qiagen, Valencia, CA) according to the manufacturer's instructions, and cDNA was synthesized from 2 μg of total RNA by reverse transcriptase (Superscript II Preamplification System; Gibco**-**BRL, Gaithersburg, MD). Real-time polymerase chain reaction (PCR) was performed on an ABI Prism 7500 sequence detection system using a SYBR Green PCR Master Mix (Applied Biosystems, Foster City, CA) and following the manufacturer's protocols. The ABI 7500 sequence detector was programmed with the following PCR conditions: 40 cycles of 15-s denaturation at 95 °C and 1-min amplification at 60 °C. All reactions were run in triplicate and normalized to the housekeeping gene β-actin. The evaluation of relative differences of PCR results was calculated by using the comparative cycle threshold (C_T_) method. The following primer sets were used:

mouse *Cxcl9* forward, 5′- GCACGATCCACTACAAATCCC-3′, and reverse, 5′- TCCGGATCTAGGCAGGTTTG-3′; mouse *Cxcl11* forward, 5′- GGAAGGTCACAGCCATAGCC-3′, and reverse, 5′- GATCTCTGCCATTTTGACGGC-3′ (Fig. 1).

mouse *Tnfα* forward, 5′-GACCCTCACACTCAGATCATCTTCT-3′, and reverse, 5′-CCTCCACTTGGTGGTTTGCT-3′; mouse *Il6* forward, 5′-GTCCTTCCTACCCCAATTTCC A-3′, and reverse, 5′-GGATGGTCTTGGTC mouse *Tnfsf11* forward, 5′-TGGAAGGCTCATGGTTGGAT-3′, and reverse, 5′-CATTGATGGTGAGGTGTGCA-3′ (Fig. 2).

mouse *Actb* forward, 5′-ATGTGGATCAGCAAGCAGGA-3′, and reverse, 5′-AAGGGTGTAAAACGCAGCTC- 3′.

### 2.4 Statistical analysis

Data are presented as the mean. Statistical analysis was performed by unpaired, two-tailed Student's *t* test using GraphPad Prism 5.0 (GraphPad Software, San Diego, CA). Values of *P* < 0.05 were considered significant.

## References

[1] Lacotte S., Brun S., Muller S., Dumortier H. (2009). CXCR3, inflammation, and autoimmune diseases. Ann. N.Y. Acad. Sci..

[2] Lee J.-H., Kim B., Jin W.J., Kim H.-H., Ha H., Lee Z.H. (2017). Pathogenic roles of CXCL10 signaling through CXCR3 and TLR4 in macrophages and T cells: relevance for arthritis. Arthritis Res. Ther..

[3] Kim B., Lee J.-H., Jin W.J., Kim H.-H., Ha H., Lee Z.H. (2018). JN-2, a C-X-C motif chemokine receptor 3 antagonist, ameliorates arthritis progression in an animal model. Eur. J. Pharmacol..

[4] Jin W.J., Kim B., Kim D., Park Choo H.-Y., Kim H.-H., Ha H., Lee Z.H. (2017). NF-kappaB signaling regulates cell-autonomous regulation of CXCL10 in breast cancer 4T1 cells. Exp. Mol. Med..

[5] Kwak H.B., Ha H., Kim H.-N., Lee J.-H., Kim H.S., Lee S., Kim H.-M., Kim J.Y., Kim H.-H., Song Y.W., Lee Z.H. (2008). Reciprocal cross-talk between RANKL and interferon-gamma-inducible protein 10 is responsible for bone-erosive experimental arthritis. Arthritis Rheum..

